# Robots and cyborgs: to *be* or to *have* a body?

**DOI:** 10.1007/s10202-012-0107-4

**Published:** 2012-05-30

**Authors:** Emma Palese

**Affiliations:** Department of Classical Philology and Philosophical Sciences, Università del Salento, Lecce, Italy

## Abstract

Starting with service robotics and industrial robotics, this paper aims to suggest philosophical reflections about the relationship between body and machine, between man and technology in our contemporary world. From the massive use of the cell phone to the robots which apparently “feel” and show emotions like humans do. From the wearable exoskeleton to the prototype reproducing the artificial sense of touch, technological progress explodes to the extent of embodying itself in our nakedness. Robotics, indeed, is inspired by biology in order to develop a new kind of technology affecting human life. This is a bio-robotic approach, which is fulfilled in the figure of the cyborg and consequently in the loss of human nature. Today, humans have reached the possibility to modify and create their own body following their personal desires. But what is the limit of this achievement? For this reason, we all must question ourselves whether we *have* or whether we *are* a body.

Nowadays, the presence of robots in our society is increasing and the same criteria for a classification of robotics are multiple and often questionable. Traditionally, we can distinguish between industrial and service robotics. It is a distinction which is, nowadays, more of a historical value than of a substantial one. For industrial robotics, in fact, it is common to identify manufacturing applications mostly carried out with manipulators (anthropomorphically or not), which have been adopted with the primary aim of helping man in repetitive, heavy, and dangerous tasks. The success of this type of applications is related to the effectiveness of the robotic production in terms of performance (accuracy, repeatability, and reliability) and, therefore, of economy. The term of service robotics is used to identify systems of mobile robots such as land, marine, aeronautical, or space vehicles equipped with some forms of autonomy in managing their own motion or sensory systems. The applications of service robotics include surveillance, collection of environmental information, logistics, and domestic tasks such as mowing the lawn in the garden or cleaning the floors of the house. The diversity and pervasiveness of the robots in our daily lives are, however, not limited to certain traditional contexts of industrial and service robotics. The robotics research of recent decades has advanced in different directions: one is that of autonomy, that is, by developing systems capable of dealing in environments not completely structured and known in advance (such as, for example, a cell of industrial production) by delegating to the robot the ability to “decide” when unforeseen situations arise. In this context, the results are remarkable, as evidenced by, for example, the success of the realization of prototype cars that can drive autonomously on roads for very long distances or in the development of teams of underwater robots showing “cognitive” abilities in navigation and guidance. We can talk about abilities exceeding the figure of the robot, that is, the cyborg (Palese 2011), because in our contemporary world, the advanced scientific progress combines the artificial reality with the human and the natural one, as the technique is embodied in each of us and thus becomes an extension of the body. It is not only the way, in which the artificial element is presented, that changes, but also the relationship between technology and humankind. This relationship incorporates in itself an extensive material that is not a simple supplement of Gehlen’s (1990) instinctual lack, but is the beginning of the transformation of the body and its way of unleashing. The body exists and widens in the world with the artificial dimension. As a result, the artificial intelligence seems to coincide with the existence, which becomes the place where the characteristic feature is the absence of nature as technology rather than simply besieging the outside, technology has installed itself in our members. Therefore, today, the classical view is disappearing, namely that the technique would be a supplement to a nature lacking in something, since the artificial, now, coincides with our being naked and with our body in which continuous technology flows are embodied. The bodies are nothing but open dimensions to contact, exposed to an otherness that radiates up to make the body coincides with technology. It introduces itself as “body given, multiplied, multisexed, multifigured, multizoned, phallic and aphallic, cephalic and acephalic, organized and inorganic” (Nancy 2007). The body, therefore, incorporates and adopts a continuous and constant metamorphosis, and man becomes a union between artificial and biological creation in which nature and culture finally find their meeting point, cancelling each other in favor of a neighbor as “téchne”—creation, the true art of our world. The whole life is resolved into a set of technical reports and technical conditions, which are the matrices of an “ecotecnia that creates the world of bodies” (Nancy 2007). These bodies seem to be totally eradicated from any possibility of being absolutely and completely immersed in the dynamic flow and a continuous flux in their changing. Transformation and transmutation are the attributes that determine both the way of *being* or *having* a body (Fromm 1976) in our contemporary age in which we see the overlap or rather the perfect coincidence between technology and nature, body and machine. This fusion between technology and the human body can have a positive and a negative meaning. In the positive meaning, technique is presented as a substantial imitation of natural biological processes and, therefore, as man’s constant desire to stay alive, to get back into life, in his bodily dimension. In this case, the artificial element becomes one with the natural rhythm of the body as it follows each performance mode, every way to perform the natural bodily function. Two distinct forces, natural and artificial, are united not as compensation but as incorporation and embodiment. The technique embodies and becomes itself the body which escapes the fate, evolving in anti-fate, since man presents himself as being able to choose, to self-produce and regenerate, following, however, the map of the natural flow of life. For instance, a pacemaker implanted in the body is a machine which is embodied as it participates in the functions of the body, allowing the heart to normalize its beat, to return to its natural rhythm. A device, therefore, that joins the body, thanks to its biocompatibility. It is compatible with the body, which means that it is a natural element for it. A technique that makes you one with nature in the “compassion”—which stems from “cum patior”—in the suffering together, for which the artificial element is joined to the natural. Moreover, we can consider the events of 2010 when the first permanent artificial heart transplant took place in Rome and the double hand transplant in Monza. These examples help us to understand how technological development can replace human body parts and save humans from their fate. It is a robotic technology inspired by biology and related to biomedical applications. The studies of bio-mechanical and bio-mimetic robotics have led, for example, to the realization of prosthesis arts, which are increasingly sophisticated and effective and may be interfaced with the nervous system of the user. Similar technologies have led to the development of exoskeletons that can help people in their mobility. Then, there is the frontier of the so-called neuromorphic technologies (Neuromorphic Engineering and Neuroinformatics) that are involved in the study and realization of artificial systems designed on the basis of studies on human or animal physiology. The results in this context are of great interest not only in terms of basic research, but certainly also for applications that include, for example, the artificial retina which, hopefully, will restore sight for some people 1 day.

However, if acting on the body requires to possess it as one thing that occurs to make a choice, a desire, a taste, it means that through the manipulation of the body, we are not only in the realm of *having,* but we also choose of *being.* In shaping the body you have, you can model it depending on what you want to be like so that in such a paradigm it becomes what you want to appear like. Here, the being becomes mere appearance, losing all the ways of true determination and proper speaking; man is not a being but a “becoming” in perpetual change, since he can become all and recreate himself as he likes to. Thus, in our contemporary world, to *have* is gobbled up by to *become* and, consequently, man loses his essence and remains only with appearance.

Just the appearance is based on the consideration of the body as the wrapper with contingent and accessorial qualities, changing over time and lacking of internal. To *be* and to *have* take on a relationship of mutual interconnection, both relying on the rejection of the body as a primary branch of life and as the ultimate expression of the generative process of nature. *Having* a body means, in fact, to reify it, to manipulate it according to its own voluntary rational force. *Being* a body means to build and realize our individuality through attributes that are assigned and which characterize the being according to the Frommian definition of “copula”, “that is, as grammatical denotation of identity” (From 1976), quite different from being as an essence. To *be* a body should, therefore, assume the value of an existence which considers man as having an essence and a very precise and specific nature. That goes beyond that being, meant as copula, arising from the consideration of the body as entity with countless accessories determining the identity of a subject. Hence, to exist as a body can have a negative and a positive sense. In the positive meaning, *being a body* makes us consider an entire and unique being presupposing a certain sacredness of what we are. In the negative meaning, however, *being a body,* meant as a determination of the individuality of a man through his physical characteristics, means to join *being* with *having being* and consider the body as something through which to appear in the contingency of the human condition. The manipulability of the body, in fact, puts the man into an existence, which, by becoming one with the historical flow, acquires an acute sense abandoning the natural, biological, and vital dimension. Just at this temporality of the dimensions of present–past–future, we see the perspectivism of Dilthey (2012), asserting that man, endowed with reason and will, descends into a kind of historical self-production which determines him as an actor, able to manage the body that he is and he has. Man thus considers himself outside of his simple life, in the sphere of what makes him human, that is, being in the existence, a situation based on the individual’s knowledge of the self but also the knowledge of the non-self, making it being that may even be different than himself. The human being changes and becomes a person that makes him by himself. His body, its physical elements, seems to insert into a sphere of substantive subordination to their choices, their capacity and ability to change, of mutation and manipulation. The existing body thus becomes the creation of man himself, his real essence, which does not arise from human nature, but from the rationality of human action, giving culture the primacy over nature since existing assumes a central role compared to living. The biological body is, therefore, regarded as a pure substrate on which to exercise their sovereignty, becoming an “object body”. However, the action of a rational human being on their own animality is nothing but the estrangement of human beings from themselves, denying the body as a point of radiation and derivation of existence, deprived of the natural flow of life. Indeed, it seems that the very existence acquires a significant value through the separation from life that, abandoning its natural and organic state of being, assumes human, anthropological, cultural, and historical characteristics (Parsi 1998). It is only in the historical self-production that man strives to increase his potentiality and self-generation. In addition to making explicit a complete distrust of modern man against the natural processes, this self-generation seems to be a mode of elevation and determining the human which, taking the distances from animality, increasingly acquires his own existential characterization (Bauman 1998). Therefore, the terms *poiesis* and *genesis* have become, by now, inseparable, and their substantial interchangeability has inevitably led to a *poiesis naturans,* that is, toward a complete substitution of human production of natural generation. Man, therefore, becomes an individual existing, being purged from his animality because he can control, manage, and direct it. And in order to manage the biological life, it is necessary to identify the origin of itself, which, in this case, can be nothing but the body from which the most authentic natural meaning of life is being disclosed. Thus, in the technological age, par excellence, the body returns to be the object (Bauman 2005) the battlefield and the meeting point on which the post-modern human beings challenge themselves imposing their beloved voluntary rational character on those humiliating and “indifferent” biological mechanisms belonging to the body and to the living organism. The technological man, therefore, appears as being infinitely a creator of himself and able to improve, enhance, and self-correct the body he has, he is and by which he appears (Bauman 2010).

When we think of *having* a body, we come to the individual appropriation of it and when we consider to *be* a body, we do nothing but open the doors to political reappropriation of our body, because a process of individualization is triggered by it. Even being a body is now determined through a mechanism of individualization and subjectivation (Rousseau 1762). An example, in this sense, is the case with the Nazi experience when it was empirically thought to be not a policy of the body, but a policy on life, on the bodies, that is, a thanatopolitics (Figiani 2008). It is nowadays present every day wherever human life is conceived outside of man himself, legitimizing the rational-voluntary domain on the animal-biological one. Moreover, we could easily argue that totalitarianism and liberalism assume a common denominator, namely the mastery of man over his animal nature, in that, if for Nazism, man is his own body, and only it, for Liberalism, according to Locke (2010), man has and owns his own body—and therefore he can use, transform, or sell it as a domestic slave. Thus, the conceptual categories of liberalism overturn the Nazist perspective (Arendt 1998) transferring the ownership of the body from state to individual. This means that, when we think we *have* a body, we come to the individual appropriation of it and, when we think of *being* a body, we do nothing but open the doors to political reappropriation of our body, because this condition starts a process of individualization and it ceases to be a natural essence of man (De Nardi 1999).
